# A Transparent Window into Biology: A Primer on *Caenorhabditis elegans*

**DOI:** 10.1534/genetics.115.176099

**Published:** 2015-06-03

**Authors:** Ann K. Corsi, Bruce Wightman, Martin Chalfie

**Affiliations:** * Biology Department, The Catholic University of America, Washington, DC 20064; † Biology Department, Muhlenberg College, Allentown, Pennsylvania 18104; ‡ Department of Biological Sciences, Columbia University, New York, New York 10027

**Keywords:** *C. elegans*, nematodes, Primer, single-cell analysis, transparent genetic system

## Abstract

A little over 50 years ago, Sydney Brenner had the foresight to develop the nematode (round worm) *Caenorhabditis elegans* as a genetic model for understanding questions of developmental biology and neurobiology. Over time, research on *C. elegans* has expanded to explore a wealth of diverse areas in modern biology including studies of the basic functions and interactions of eukaryotic cells, host–parasite interactions, and evolution. *C. elegans* has also become an important organism in which to study processes that go awry in human diseases. This primer introduces the organism and the many features that make it an outstanding experimental system, including its small size, rapid life cycle, transparency, and well-annotated genome. We survey the basic anatomical features, common technical approaches, and important discoveries in *C. elegans* research. Key to studying *C. elegans* has been the ability to address biological problems genetically, using both forward and reverse genetics, both at the level of the entire organism and at the level of the single, identified cell. These possibilities make *C. elegans* useful not only in research laboratories, but also in the classroom where it can be used to excite students who actually can see what is happening inside live cells and tissues.

*Here, for the first time, GENETICS and WormBook, the online review of C. elegans biology, co-publish an article. As mission-driven, community publishers, we seek to provide the most widely accessible resource available to researchers. We wish to thank Jane Mendel, WormBook Editor, for her dedication to this collaboration, and Marty Chalfie for his vision*.

IN 1963, Sydney Brenner sent a letter to Max Perutz, the chairman of the Medical Research Council’s Laboratory of Molecular Biology (LMB), detailing his concerns that the “classical problems of molecular biology have either been solved or will be solved in the next decade” and proposing that the future of molecular biology lies in the extension to other fields, “notably development and the nervous system” (Brenner 1988, 2002). With the simplicity and power of prokaryotic genetics in mind, he proposed that a nematode (round worm), *Caenorhabditis briggsae*, would be an ideal system in which to tackle these problems. Later, he settled on the related nematode *C. elegans* as the focus of his efforts because the *elegans* strain grew better than the *briggsae* isolate in Brenner’s laboratory (Félix 2008). Today, *C. elegans* is actively studied in over a thousand laboratories worldwide (www.wormbase.org) with over 1200 *C. elegans* research articles published each year for the last 5 years.

*C. elegans* is a tiny, free-living nematode found worldwide. Newly hatched larvae are 0.25 mm long and adults are 1 mm long. Their small size means that the animals usually are observed with either dissecting microscopes, which generally allow up to 100X magnification, or compound microscopes, which allow up to 1000X magnification. The dissecting microscope is used to observe worms on Petri dishes (Figure 1, A and B) as they move, eat, develop, mate, and lay eggs (for movies showing these features, see http://labs.bio.unc.edu/Goldstein/movies.html). A compound or confocal microscope allows observation at much finer resolution (Figure 1C), permitting researchers to perform experiments that address questions related to cell development and function at single-cell resolution. Because *C. elegans* is transparent, individual cells and subcellular details are easily visualized using Nomarski (differential interference contrast, DIC) optics (Figure 1C). Enhanced detail can be discerned by using fluorescent proteins to tag proteins or subcellular compartments (Figure 1D). Fluorescent proteins can also be used to study developmental processes, screen for mutants affecting cell development and function, isolate cells, and characterize protein interactions *in vivo* (Chalfie *et al.* 1994; Boulin *et al.* 2006; Feinberg *et al.* 2008).

**Figure 1:**
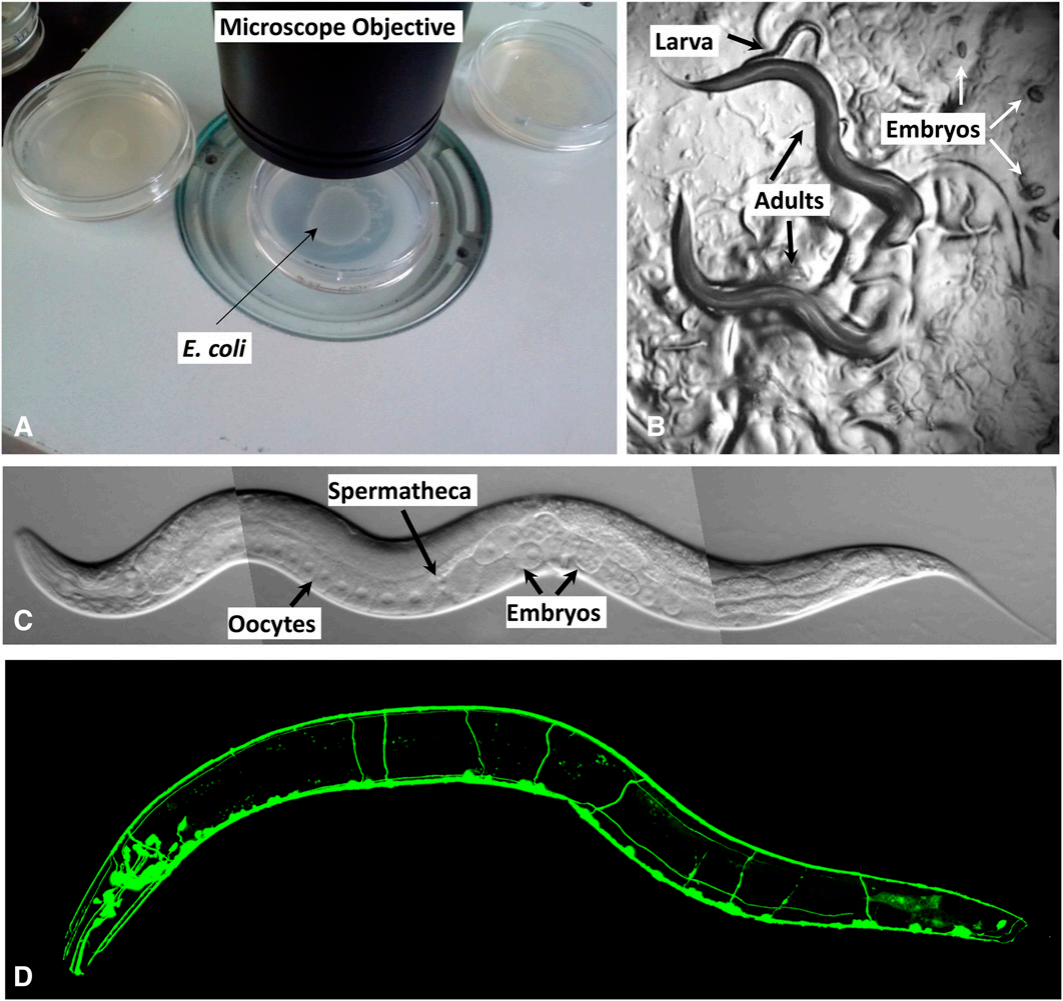
Observing *C. elegans*. (A) Petri dishes sitting on the base of a dissecting stereomicroscope. Bacterial lawns are visible on the surface of the agar inside the dishes but the *C. elegans* are too small to be seen in this view. (B) *C. elegans* viewed through the dissecting microscope. The two adults are moving in this view. Tracks in the plate indicate where animals have traveled on the bacterial lawn. (C) An adult hermaphrodite is viewed in a compound microscope. In all pictures, anterior is to the left and ventral is on the bottom. *C. elegans* moves on either its left or right side; in this image the surface facing the viewer is the left side. Because the animals are transparent, one can see, from left to right on the ventral side, developing oocytes in the gonad (rectangular cells with a clear, circular nucleus inside) followed by the spermatheca (where oocytes are fertilized), and multiple embryos in the uterus. (D) Fluorescent image showing the nervous system labeled with a GFP reporter (*sto-6::gfp*). Photo credits: (C) Original (modified here): B. Goldstein; (D) J. Kratz.

*C. elegans* has a rapid life cycle (3 days at 25° from egg to egg-laying adult) and exists primarily as a self-fertilizing hermaphrodite, although males arise at a frequency of <0.2% (Figure 2). These features have helped to make *C. elegans* a powerful model of choice for eukaryotic genetic studies. In addition, because the animal has an invariant number of somatic cells, researchers have been able to track the fate of every cell between fertilization and adulthood in live animals and to generate a complete cell lineage (Sulston and Horvitz 1977; Kimble and Hirsh 1979; Sulston *et al.* 1983). Researchers have also reconstructed the shape of all *C. elegans* cells from electron micrographs, including each of the 302 neurons of the adult hermaphrodite (White *et al.* 1986) and the posterior mating circuit in the adult male (Jarrell *et al.* 2012). These reconstructions have provided the most complete “wiring diagrams” of any nervous system and have helped to explain how sexual dimorphism affects neuronal circuits. Moreover, because of the invariant wild-type cell lineage and neuroanatomy of *C. elegans*, mutations that give rise to developmental and behavioral defects are readily identified in genetic screens. Finally, because *C. elegans* was the first multicellular organism with a complete genome sequence (*C. elegans* Sequencing Consortium 1998), forward and reverse genetics have led to the molecular identification of many key genes in developmental and cell biological processes.

**Figure 2:**
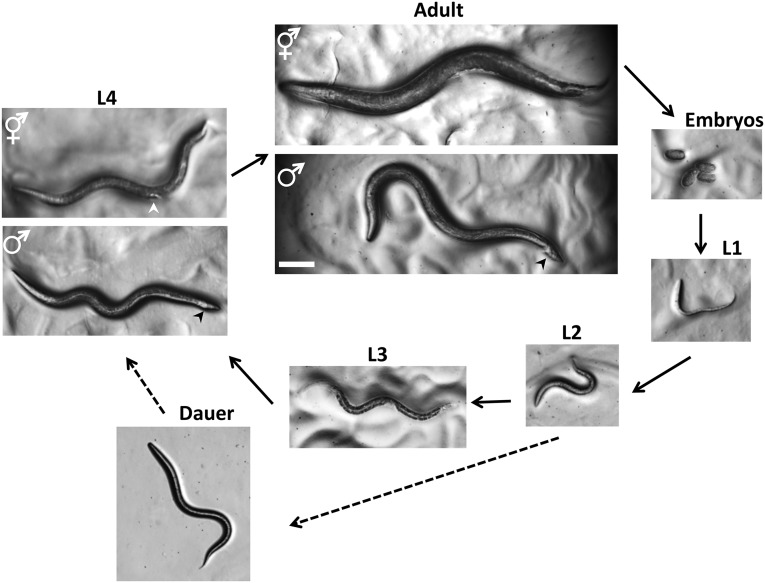
Life cycle of *C. elegans*. Animals increase in size throughout the four larval stages, but individual sexes are not easily distinguished until the L4 stage. At the L4 stage, hermaphrodites have a tapered tail and the developing vulva (white arrowhead) can be seen as a clear half circle in the center of the ventral side. The males have a wider tail (black arrowhead) but no discernible fan at this stage. In adults, the two sexes can be distinguished by the wider girth and tapered tail of the hermaphrodite and slimmer girth and fan-shaped tail (black arrowhead) of the male. Oocytes can be fertilized by sperm from the hermaphrodite or sperm obtained from males through mating. The dauer larvae are skinnier than all of the other larval stages. Photographs were taken on Petri dishes (note the bacterial lawns in all but the dauer image). Bar, 0.1 mm.

The experimental strengths and the similarities between the cellular and molecular processes present in *C. elegans* and other animals across evolutionary time (metabolism, organelle structure and function, gene regulation, protein biology, etc.) have made *C. elegans* an excellent organism with which to study general metazoan biology. At least 38% of the *C. elegans* protein-coding genes have predicted orthologs in the human genome (Shaye and Greenwald 2011), 60–80% of human genes have an ortholog in the *C. elegans* genome (Kaletta and Hengartner 2006), and 40% of genes known to be associated with human diseases have clear orthologs in the *C. elegans* genome (Culetto and Sattelle 2000). Thus, many discoveries in *C. elegans* have relevance to the study of human health and disease.

## *C. elegans* Basics

### Growth and maintenance

*C. elegans*, although often mischaracterized as a soil nematode, can most easily be isolated from rotting vegetable matter, which contains an ample supply of their bacterial food source (Barrière and Félix 2014). In the laboratory, animals are normally grown on agar plates containing a lawn of the bacterium *Escherichia coli*. Once the animals deplete the bacteria, they utilize their fat supply. Without food, the development of young larval-stage animals is arrested. As a result of entering this stasis, animals can survive for at least a month (often starved plates can be usefully kept for up to 6 months at 15°), and as stocks, they do not require constant feeding. Whenever healthy, growing animals are needed, a piece of the agar from the old plate can be transferred to a new plate with bacteria. The animals move to the new bacteria and resume their development.

Several other features greatly facilitate the maintenance of *C. elegans* stocks and their experimental use. First, because *C. elegans* is a self-fertilizing hermaphrodite (see the following section), a single animal can populate a plate. Second, animal populations can be frozen for years and revived when needed. Third, the animal’s small size means that many can be grown in a small space. Fourth, animals can be grown at temperatures ranging from 12° to 25°; their Q_10_ for growth is ∼2 (that is, an increase of 10° speeds up growth twofold). Growth at different temperatures makes it possible to control the rate of animal development and assists in the isolation and use of temperature-sensitive mutants. Continual growth above 25° is not possible because the animals become sterile. The upper temperature limit can be a problem if animals are kept on bench tops (instead of temperature-controlled incubators) in rooms that are too warm. Shorter exposures to higher temperatures are possible for heat shock experiments and to increase production of males (Sulston and Hodgkin 1988). Fifth, animals can be synchronized by isolating newly hatched larvae or by treating gravid adults with bleach (which decontaminates by killing everything but embryos) and isolating eggs, which are resistant to bleach treatment. Sixth, to facilitate biochemical studies, animals can be grown in bulk in liquid medium. “Worm sorters” such as the COPAS Biosorter are also available to quickly select large quantities of individual worms with desired characteristics. Finally, one does not need especially expensive equipment beyond a good dissecting microscope and a compound microscope to work with this animal. Overall, the animals are inexpensive and convenient to maintain.

### Sexual forms and their importance

**Figure 3:**
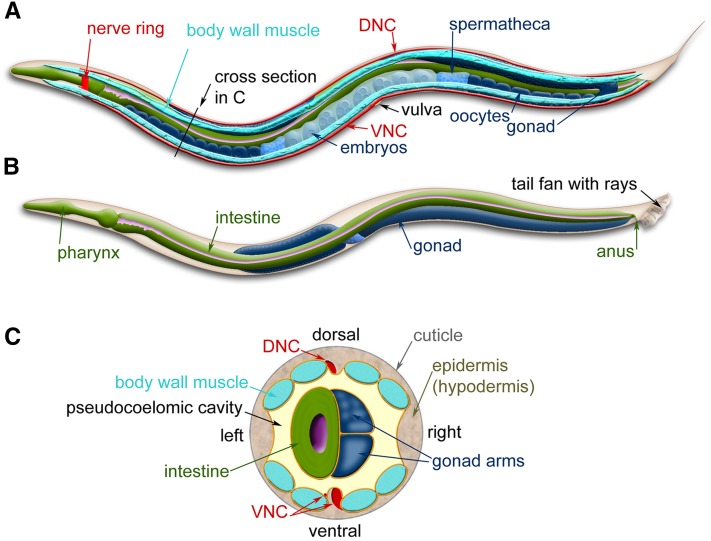
*C. elegans* anatomy. Major anatomical features of a hermaphrodite (A) and male (B) viewed laterally. (A) The dorsal nerve cord (DNC) and ventral nerve cord (VNC) run along the entire length of the animal from the nerve ring. Two of the four quadrants of body wall muscles are shown. (B) The nervous system and muscles are omitted in this view, more clearly revealing the pharynx and intestine. (C) Cross-section through the anterior region of the *C. elegans* hermaphrodite (location marked with a black line in A) showing the four muscle quadrants surrounded by the epidermis and cuticle with the intestine and gonad housed within the pseudocoelomic cavity. Images are modified from those found at www.wormatlas.org (Altun *et al*. 2002–2015).

Wild-type *C. elegans* has two sexual forms: self-fertilizing hermaphrodites and males (Figure 2 and Figure 3, A and B). The gonad of hermaphrodites forms an ovotestis that first produces haploid amoeboid sperm that are stored in the spermatheca in the L4 stage and then near adulthood the germ line switches fate to produce much larger oocytes. Essentially hermaphrodites are females whose gonads temporarily produce sperm before they produce oocytes. Hermaphrodites can produce up to 300 self-progeny that are fertilized by the stored sperm. If mated with males, hermaphrodites are capable of producing ∼1000 offspring, indicating that hermaphrodite-produced sperm is a limiting factor in self-fertilization. Both sexes are diploid for the five autosomal chromosomes. The sexes differ in that hermaphrodites have two X chromosomes and males have a single X chromosome—*C. elegans* has no Y chromosome—and the genotype of males is referred to as XO. Sex is determined by the X to autosome (X:A) ratio (Zarkower 2006). The majority of offspring produced by self-fertilization are hermaphrodites; only 0.1–0.2% of the progeny are males due to rare meiotic nondisjunction of the X chromosome. Because hermaphrodites make their own sperm, in genetic crosses self-progeny (oocytes fertilized by the hermaphrodite’s sperm) need to be distinguished from cross-progeny. For example, when hermaphrodites homozygous for a recessive mutation causing a visible mutant phenotype are mated to wild-type males, the self-progeny hermaphrodites show the mutant phenotype and the cross-progeny hermaphrodites do not.

Self-fertilizing hermaphrodites provide several advantages for genetic analysis. First, self-fertilization (often referred to as selfing) simplifies maintaining stocks because a single animal can give rise to an entire population. Second, as Brenner (1974) has written, “the animals are driven to homozygosity,” *i.e.*, populations of hermaphrodites tend to lose heterozygotes (because hermaphrodites cannot mate with other hermaphrodites). Thus, strains that are mutagenized are essentially isogenic. Third, selfing follows the standard Mendelian rules of segregation, so a parent that is heterozygous for a recessive trait will produce the standard 1:2:1 pattern of segregation, such that 25% of the progeny will be homozygous for the mutant allele and display the autosomal recessive trait. Thus, selfing reduces greatly the effort needed to find such mutants. Fourth, mutants with neuromuscular defects that impair the ability to mate can still be maintained in the laboratory. In fact, only 11 of the 302 nerve cells of the hermaphrodite: eight ADF, ASG, ASI, and ASJ neurons, which when killed as a group cause the animals to become dauer larvae (Bargmann and Horvitz 1991), the two CAN cells (Forrester and Garriga 1997), and the M4 neuron in the pharynx (Avery and Horvitz 1989) are known to be essential to support development to the reproductive adult stage. Fifth, the viability of even severely defective mutants and their ability to self-fertilize allows for easy screens for modifier (enhancer and suppressor) mutations. Such screens have been exceptionally useful and informative. For example, *lin-12* mutants (*C. elegans* nomenclature is outlined in Box 1) are defective in vulval development and components of the LIN-12/Notch signaling pathway have been identified with both suppressor and enhancer screens (Greenwald and Kovall 2013).

**Box 1. *C. elegans* Nomenclature**

**Table:** Genetic nomenclature differs from species to species. Here, we describe the major terms used in *C. elegans* research. A more complete description can be found at www.wormbase.org/about/userguide/nomenclature.

**Nomenclature at a glance**^1^**:**	
**Term**	**Definition**
ZK154.3	Systematic gene identification (3^rd^ predicted gene on cosmid ZK154)
*mec-7*	Gene name (the 7^th^ “mechanosensory abnormality” gene named)
*mec-7(e1506)*	Allele name (from the MRC Laboratory of Molecular Biology - *e*)
MEC-7	Protein name (product of *mec-7* gene)
Mec	Phenotype (Mechanosensory abnormal phenotype)
*e1506*	Homozygous allele
*e1506/+*	Heterozygous allele
*mnDp30*	Duplication (from the Herman Lab - *mn*)
*nDf6*	Deficiency/Deletion (from the Horvitz Lab - *n*)
*muIs35*	Integrated transgene (from the Kenyon Lab - *mu*)
*evEx1*	Extrachromosomal transgene array (from the Culotti Lab - *ev*)
CB3270	Strain name (from MRC Laboratory of Molecular Biology - CB)
*mec-7p::gfp*	GFP transcriptional fusion (using only the promoter of the gene)
*mec-7::gfp*	GFP translational fusion (in which *gfp* is inserted in the coding sequence of the gene)
*ceh-6(pk33::Tc1)*	Transposon (Tc1) insertion in *ceh-6* gene

^1^ All *C. elegans* gene names, allele designations, and reporter genes are written in italics. Cosmids, proteins, phenotypes, and strain names are not written in italics.

**Mutation (allele) names**: The wild-type allele of any gene is designated by an italicized plus sign, *+.* Mutant alleles are represented by 1-3 lowercase letters, which indicate specific laboratories, followed by a number. All gene and allele symbols are italicized, *e.g., unc-54*, *e678*, and *mn5*. The homozygous genotype is represented by a single copy of the allele name (*e678*). A heterozygous condition is indicated with a slash, *e.g., e364/+*, *e364/e1099.* Chromosomal abnormalities are indicated by one or two letters after the lab code, *e.g., mnDp30* is a duplication, *nDf6* is a deletion (called a deficiency), and *szT1* is a translocation. One or two letters may be added after the allele name to indicate particular properties conveyed by the mutation *e.g.* temperature-sensitivity (ts), amber (am), revertant (r), dominant (d), and semidominant (sd). When a gene has more than one mutation, such as could result from the intragenic reversion of a mutant allele, both the old and new mutations are indicated, *e.g.,* intragenic reversion of *e1498* could yield *e1498u124r*.

**Gene names**: Genes are designated by 3 or 4 lower case letters, a dash, and a number (all italicized), *e.g. lon-2* and *ensh-1*. To distinguish alleles of the same gene, the allele names are placed in parentheses with no space between the gene and allele name, *e.g., mec-7(e1343)* and *mec-7(e1506)*. Genes that have been identified as an open reading frame (ORF) through bioinformatics approaches get a systematic gene identification (*e.g.* ZK154.3) until subsequent studies prompts them to be given a gene name. The upstream promoter region of a gene is indicated by the gene name followed by a “p.” The promoter and the protein-coding names are separated by two colons as are parts of fusion proteins. Thus, a gene encoding a MEC-7::GFP translational fusion driven from the *mec-7* promoter would be *mec-7::gfp*, whereas a *mab-5* transcriptional fusion would be *mab-5p::gfp*. A transposon insertion into a gene is similarly shown using the transposon name and two colons, e.g., *unc-22::Tc1* [most *C. elegans* transposons are labeled Tc (transposon, *Caenorhabditis*) and a number].

**Phenotypes**: Mutant phenotypes are designated by the non-italicized gene name without the dash and number and with the first letter capitalized, e.g., Unc or Sma animals are Uncoordinated or Small in body size.

**Gene products**: RNAs are designated by the italicized gene name; proteins are designated by the gene name in all caps and not italicized (MEC-7). Sometimes specific amino acid changes are indicated.

**Strain names**: Two capital letters and a number, *e.g.* CB429 and TU38, designate a strain containing one or more genetic differences. The letters indicate the laboratory that constructed the strain (http://www.cbs.umn.edu/research/resources/cgc/lab-head). None of these symbols are italicized. Because a given strain can carry more than one mutation, strain names can be thought of as shorthand to describe animals carrying complicated genotypes. Genes (and/or alleles) in strains with several mutations are listed in chromosome and then map order with genes on the same chromosome separated by spaces and genes on different chromosomes separated by semi-colons. The italicized name of the chromosome can also be included, *e.g., lon-2(e678) mec-7(e1506) X*, *unc-54; myo-3*, and *e364; e66; u38/szT1*. *szT1* is one of many balancer chromosomes that are often used to maintain mutations that are lethal as homozygotes but viable as heterozygotes. For strains that contain reporter genes, there are two typical designations. The reporters are named for specific laboratories similar to mutant alleles, and they are further named according to whether the reporter gene is maintained extrachromosomally on a multicopy array using an “Ex” label (*e.g. evEx1)* or is maintained by integrating the sequence (in most cases randomly) into the genome using an “Is” label (*e.g. muIs35*).

Males are important because they allow the exchange of genetic material needed to generate animals with different genetic compositions and to map genes. Indeed, the animal has evolved to take advantage of the genetic contribution of rare males by using male (outcross) sperm before using hermaphrodite (self) sperm (Ward and Carrel 1979). Thus, if males are capable of mating, cross-progeny prevail (to help males find hermaphrodites, researchers use Petri plates with a small spot of bacteria).

### Life cycle

*C. elegans* embryogenesis takes ∼16 hr at 20° (all of the subsequent times are also for development at 20°) (Figure 2). A virtually impermeable eggshell is made after fertilization, allowing the embryo to develop completely independent of the mother. However, embryos are usually retained within the hermaphrodite until about the 24-cell stage at which time they are laid. The hermaphrodite embryo hatches with 558 nuclei (some nuclei are in multinuclear syncytia, so the cell count is lower) and becomes a first stage (L1) larva. The animals begin to eat and develop through four larval stages (L1–L4). The L1 stage is ∼16 hr long; the other stages are ∼12 hr long. Each stage ends with a sleep-like period of inactivity called lethargus (Raizen *et al.* 2008) in which a new cuticle (outer collagenous layer) is made. Lethargus ends with the molting of the old cuticle. Approximately 12 hr after the L4 molt, adult hermaphrodites begin producing progeny for a period of 2–3 days until they have utilized all of their self-produced sperm; additional progeny can be generated if the sperm-depleted hermaphrodite mates with a male. After the reproductive period, hermaphrodites can live several more weeks before dying of senescence.

When bacteria are depleted and the animals are crowded, L2 larvae activate an alternative life cycle (Hu 2007) and molt into an alternative L3 larval stage called the “dauer” larva (“dauer” in German means “lasting”; the signal is actually processed by L1 animals, but its results are not seen until the so-called “L2d” stage; Golden and Riddle 1984). The dauer larva cuticle completely surrounds the animal and plugs the mouth preventing the animal from eating and thereby arresting development. The dauer cuticle has enhanced resistance to chemicals, so it provides the dauer with greater protection against environmental stresses and caustic agents. Dauer larvae can survive for many months and are the dispersal form most commonly encountered in the wild. When the dauer larvae are transferred onto plates with bacteria, they shed their mouth plugs, molt, and continue their development as slightly different L4 larvae.

## *C. elegans* Genetics

A major reason Brenner (1974) chose to study *C. elegans* was the ease of genetic manipulation. Self-fertilization means that after hermaphrodites (P0s) are mutagenized, any mutant alleles (except dominant lethals) can be maintained through self-propagation in first-generation (F1) progeny, and second-generation (F2) progeny, etc. without mating. This property makes obtaining these progeny easy. In practice, *C. elegans* researchers screen for mutations anywhere in the genome (instead of using balanced strains to mutagenize single chromosomes) in a single mutagenesis and determine linkage afterward. A second genetic advantage of *C. elegans* is that it grows quickly. Since animals take ∼3 days at 25° (∼3.5 days at 20°) to develop from fertilized eggs to adults producing their own fertilized eggs, mutant homozygotes can be detected two generations (∼1 week) after mutagenesis. In addition, the ability to freeze and recover *C. elegans* makes it possible to preserve mutant strains without worrying that they have acquired unwanted suppressors, other modifiers, or additional background mutations or have lost important mutations, particularly if they are maintained as heterozygotes. Therefore, much less effort needs to be devoted to strain maintenance.

The traditional use of genetics in *C. elegans* (often referred to as “forward genetics”) begins with a screen or selection to find mutants with a particular phenotype followed by inference of the wild-type role of the gene from the nature of the mutant phenotype. Jorgensen and Mango (2002) have reviewed the many types of screens and selections used in *C. elegans* to identify mutations resulting in novel phenotypes (including conditional phenotypes) and mutations that modify (either enhancing or suppressing) existing phenotypes. A variety of mutagens have been used (Kutscher and Shaham 2014), including ethane methylsulfonate (EMS), an alkylating reagent that causes principally GC-to-AT transition mutations and small deletions, used by Brenner in his initial studies (Brenner 1974).

Once mutant strains have been obtained and shown to be true-breeding, *i.e.*, give mutant individuals in the next generation, they can be mapped using classical genetic tools (Brenner 1974). Originally, mapping involved linkage crosses to identify the chromosome containing the mutation followed by multiple three-factor crosses to refine its map position. Once a map position had been determined, the mutated gene could be identified molecularly (using the physical map of overlapping genomic clones) by transformation rescue of the mutant phenotype by the wild-type gene (Evans 2006; Merritt *et al.* 2010; Schweinsberg and Grant 2013). Transformation requires injection of DNA into the syncytial (multinucleated) gonad, where it is incorporated into the nucleus of some developing gametes (Mello and Fire 1995). Transposon-tagged mutations were also used to isolate the DNA of the mutated gene, allowing for its molecular identification (*e.g.*, Greenwald 1985). The entire process of cloning a gene could easily take a year.

Today, however, the process of connecting a mutant phenotype to a gene is much more rapid due to advances in whole-genome sequencing (Doitsidou *et al.* 2010; Zuryn *et al.* 2010; Minevich *et al.* 2012). Mutants derived from the standard wild-type strain (called N2) are crossed to a wild-type strain obtained in Hawaii (CB4856), whose sequence differs from N2 at many positions [one single nucleotide polymorphism (SNP) per 91 ± 56 kb; Swan *et al.* 2002]. Mutants reisolated after the cross have retained N2 sequences in the vicinity of the mutation, whereas N2 and Hawaiian sequences randomly segregate at other loci. Whole-genome sequencing can reveal the location of N2 DNA and lead, hopefully, to a small number of candidate genes that can be tested as above. This process can be done in a number of weeks and, as before, transformation rescue (or complementation testing) provides evidence that the correct gene has been identified.

Researchers can also use a known gene sequence to obtain mutant strains, a process called “reverse genetics” (Ahringer 2006). One of the first ways to do such reverse genetics was the generation of strains that deleted all or part of a target gene (Lesa 2006). Animals were mutagenized with trimethylpsoralen to cause deletions and large numbers of individual F2 animals were used to establish lines. Pooling DNA from these strains and amplifying the several pools of DNA with PCR primers, designed to amplify a deleted but not wild-type gene, could identify which line had a deletion mutation (a process called sibling selection). Further selection within the line could establish a homozygous strain containing the deletion mutation.

Whole-genome sequencing has enabled a very rapid way of obtaining mutations. In a study dubbed the “million mutation project” (Thompson *et al.* 2013), animals were mutated by EMS and/or N-ethyl-N-nitrosourea (ENU) and F2 progeny (from different P0 parents) were grown. Over 2000 healthy strains were established from these mutageneses. Whole-genome sequencing of each strain showed that, on average, there were 400 mutations per strain. Altogether these strains had over 800,000 unique mutations. Since the genome contains ∼20,000 genes, each gene has been mutated an average of eight times. The results are available in WormBase and the strains can be obtained from the *Caenorhabditis* Genetics Center (Table 1). Recent advances in efficient genome-editing methods (TALEN and CRISPR/Cas9) in *C. elegans* now allow investigators to create targeted mutations in nearly any location in the genome in any genetic background (Frøkjær-Jensen 2013; Waaijers and Boxem 2014). These gene editing techniques, particularly CRISPR/Cas9, enable rapid methods to mutate and interrogate *C. elegans* genes, and new approaches based on them are being introduced.

**Table 1:** *C. elegans* resources

Resource	Website Address	Description
		
WormBase	http://www.wormbase.org/	Genes, expression, resources, phenotypes, metadata, and publications
WormBook	http://www.wormbook.org/	Basic information about the biology of *C. elegans* and other nematodes, including methods
WormAtlas	http://www.wormatlas.org/	Worm anatomy, including neurons and wiring, EM sections, and cell lineage
*Caenorhabditis* Genetics Center	https://www.cbs.umn.edu/research/resources/cgc	Stocks of wild-type and mutant nematode strains
National Bioresource Project	http://www.shigen.nig.ac.jp/c.elegans/index.jsp	Collection of deletions of *C. elegans* genes
Million Mutation Project	http://genome.sfu.ca/mmp/	Fully sequenced genomes of strains carrying multiple mutations
Expression patterns database	http://gfpweb.aecom.yu.edu/index	Expression patterns for promoter::gfp transgenes
TransgeneOme	https://transgeneome.mpi-cbg.de/transgeneomics/index.html	Resource for tagged gene constructs and expression patterns
modENCODE	http://www.modencode.org	Model organism database of DNA elements
UCSC Genome Browser	https://genome.ucsc.edu/	Multiple alignments of conserved nematode genome sequences
*C. elegans* Interactive Network	http://www.wormweb.org/	Interactive neuron wiring diagram and gene expression information with direct links to published supporting data
OpenWorm Science	http://www.openworm.org/	Various on-line resources for *C. elegans* research.
*C. elegans* Behavioral Database	http://wormbehavior.mrc-lmb.cam.ac.uk/	Detailed traces of worm movement and posture for different strains and mutants
*Caenorhabditis* *briggsae* Research Resource	http://www.briggsae.org/	Resource for research on *C. briggsae*
Nematode and Neglected Genomics	http://www.nematodes.org/	Database of genomics for other nematode species
Rhabditina Taxonomy	http://128.122.60.136/fmi/iwp/cgi?-db=RhabditinaDB&-loadframes or http://wormtails.bio.nyu.edu/Databases.html	Nematode phylogeny, morphology, literature, ecology, and geographical information
WormClassroom	http://wormclassroom.org/	Resource for education using *C. elegans*

Mutant-like phenotypes can also be obtained using RNA interference (RNAi), the use of double-stranded RNA (dsRNA) to reduce gene activity (Fire *et al.* 1998, see Ahringer 2006). The added discovery that RNAi can be produced by soaking the animals in a solution of dsRNA (Tabara *et al.* 1998) or by feeding the animals bacteria that generate specific dsRNA (Timmons and Fire 1998) means the entire genome can be easily and systematically interrogated for genes needed for specific functions. Such genome-wide screens using libraries of bacterial strains are common and easily done.

## Why Choose *C. elegans*?

In addition to being a powerful system for genetic studies, *C. elegans* has many inherent advantages as a model for eukaryotic biology. These features include its small size, large brood size, ease of cultivation, low maintenance expense, long-term cryopreservation, quick generation time, transparency, invariant cell number and development, and the ability to reduce gene activity using feeding RNAi. Although not usually mentioned, another favorable feature of *C. elegans* is the organisms are quite benign to humans. In fact, because they cannot grow at body temperature, they cannot grow in humans. Some nematodes, *e.g.*, *Ascaris suum*, induce a debilitating allergic reaction and must be studied in ventilated hoods (Kennedy 2013; A. O. Stretton, personal communication). As far as we are aware, allergic reactions to *C. elegans* have not been documented.

**Figure 4:**
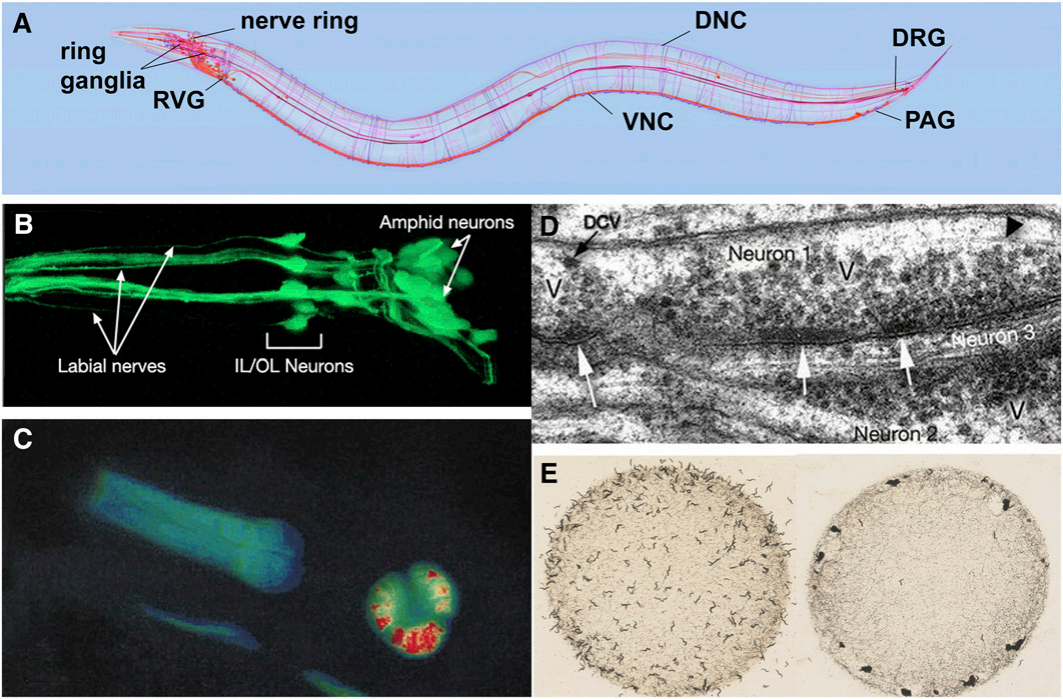
Anatomy and study of the *C. elegans* nervous system. (A) Diagram of the *C*. *elegans* nervous system identifying some major nerve bundles and ganglia. Major nerve tracts include the ventral nerve cord (VNC), dorsal nerve cord (DNC), and nerve ring. Major ganglia include the ring ganglia, retrovesicular ganglion (RVG), preanal ganglion (PAG), and dorsal-root ganglion (DRG). Image was produced using the OpenWorm browser utility (openworm.org). (B) Visualization of anterior sensory neurons and their neurite projections by expression of a GFP reporter transgene. The *Y105E8A.5::GFP* fusion transgene is expressed in amphid, OL, and IL sensory neurons of the head (R. Newbury and D. Moerman, Wormatlas; wormatlas.org). (C) Use of cameleon reporter transgene to detect calcium transients in the *C. elegans* pharynx. The animal carries a transgene with *myo-2*, a pharynx-specific myosin gene, fused to YC2.1, a calcium-sensitive fluorescent detector. False-color red in the pharyngeal bulb reflects real-time calcium releases in the cell of the living animal. Image was adapted from Kerr *et al.* (2000). (D) Electron microscopic section showing synapses. Collections of densely staining vesicles can be seen in neuron 1 at the point of synaptic connection to neurons 2 and 3 (arrows). Synaptic varicosities (V) that contain vesicles can be seen clustered around the active zone. DCV identifies a dense-core vesicle. Image is from D. Hall (Wormatlas; wormatlas.org). (E) Worm behavior on a bacterial plate. Left image shows the standard laboratory N2 strain foraging as individuals evenly dispersed across the bacterial food. Right image shows an *npr-1* mutant strain foraging in grouped masses (sometimes called a “social” feeding phenotype). Image is from M. de Bono, taken from Schafer (2005).

Studies of cell and developmental biology that use *C. elegans* are greatly aided by the transparency of the animal, which allows researchers to examine development and changes due to mutations or altered environments at the level of a single, identified cell within the context of the entire living organism. Thus, many biological problems can be studied “in miniature” at the single-cell level, instead of in large numbers of cells in heterogeneous tissues. Transparency also enables a wealth of studies in living animals utilizing fluorescent protein reporters (Figure 1D and Figure 4B). By labeling cells and proteins in living cells, fluorescent proteins enable genetic screens to identify mutants defective in various cellular processes. In addition, fluorescent protein-based reporters (*e.g.*, Cameleon and gCaMP3; Figure 4C), which fluoresce in response to calcium flux, provide neuron-specific detection of calcium flux under a fluorescent microscope and therefore allow researchers to measure electrophysiological activity *in vivo* (Kerr 2006). Furthermore, mapping of cell–cell and synaptic contacts can be accomplished by expressing complementary fragments of GFP in different cells (GRASP; Feinberg *et al.* 2008). Transparency also means that optogenetic tools, which alter the activity of individual neurons, are particularly effective in *C. elegans* (Husson *et al.* 2013). In all of these experiments, greater control of the animal’s position and environment can be accomplished by microfluidic devices in which individual worms are mounted in custom-designed channels allowing the application of various compounds or other agents while simultaneously monitoring fluorescent readout of gene regulation or electrophysiological activity by microscopy (Lockery 2007; San-Miguel and Lu 2013).

Sharing large amounts of genetic and cellular information has been central to the success of *C. elegans* research. The wealth of knowledge generated by past research is readily available to anyone via on-line resources (see Table 1). Much of this information, including gene expression, gene function, and references, is curated on WormBase (www.wormbase.org). Reviews on many topics of *C. elegans* biology are provided by WormBook (www.wormbook.org), which includes a collection of *C. elegans* methods (WormMethods) and current and past issues of the *C. elegans* Newsletter (*The Worm Breeder’s Gazette*).

No model organism can be used to answer every research question, and working with *C. elegans* has some limitations. Not all metazoan genes are found in the *C. elegans* genome (Ruvkun and Hobert 1998). For example, Hedgehog (Hh) signaling is important in vertebrates for the patterning of various organs during development, but *C. elegans* lacks many of the genes in the regulatory cascade (Bürglin and Kuwabara 2006). Although some *C. elegans* cells can be studied *in vitro* [*e.g.*, embryonic glial cells, larval muscle, and neuronal cells (Zhang *et al.* 2011; Stout and Parpura 2012)], no *C. elegans* cell culture lines exist. The small size of the animal and its cells also provides a challenge, since experimental manipulation in individual tissues of an organism that is less than a millimeter long is difficult. Electrophysiology of *C. elegans* neurons and muscle is possible, but demanding (Raizen and Avery 1994; Lockery and Goodman 1998; Cook *et al.* 2006; Richmond 2006) and indirect measurements of neuronal activity, such as calcium imaging are often used instead (Kerr 2006). Finally, biochemical approaches in *C. elegans* have lagged behind the genetic approaches, but the development of an axenic culture medium for *C. elegans* (*e.g.*, Rao *et al.* 2005) has meant that biochemical studies can be done on animals under defined conditions.

## *C. elegans* Tissues

One attractive feature of *C. elegans* is that despite its simplicity, it has defined tissues. The animal is often described as a series of concentric tubes (Figure 3C). The outer layer of cells, the epidermis (traditionally called the hypodermis) encloses a pseudocoelomic fluid-filled cavity housing the main organ systems. Just inside the epidermis are the bands of muscle, which control movement of the organism, as well as the ventral and dorsal nerve cords that innervate the muscles. Inside the neuromuscular region are the digestive, excretory, and reproductive systems. In addition, *C. elegans* has six cells in the pseudocoelomic cavity, called coelomocytes, which act as scavengers in the body cavity (Grant and Sato 2006). These cells behave similarly to vertebrate macrophages (although they have a fixed position within the animal), are highly active in endocytosis, and are thought to sort through and clear material in the pseudocoelomic cavity of the animal. More extensive descriptions of these systems with beautiful electron micrographs of the various cell types can be found in WormAtlas (www.wormatlas.org). Here, we will cover some of the basic aspects of the anatomy, particularly with respect to how the organ systems are advancing the understanding of cell and developmental biology.

### Epidermis: a model for extracellular matrix production, wound healing, and cell fusion

**Figure 5:**
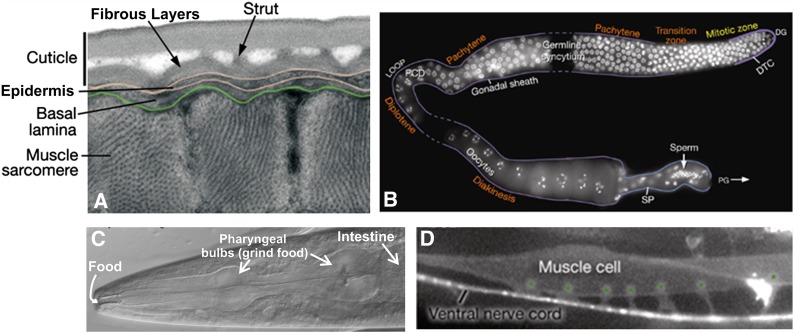
*C. elegans* tissue morphology. (A) Cross-section of the outer layers of the animal showing muscle cells below the epidermis and cuticle viewed by transmission electron microscopy. (B) Single gonad arm dissected out of a hermaphrodite showing germ cell DNA (stained white). Meiosis begins in the region labeled “pachytene” (top right) and continues around the loop of the gonad until oocytes are formed. The stored sperm are located in the spermatheca of the gonad (bottom right). This image is a composite of three gonad arms and dashed lines represent regions not captured in the individual micrographs. (C) The anterior of the animal showing the mouth where food enters, the pharynx with its two bulbs, and the beginning of the intestine viewed with differential interference contrast (DIC). (D) A single body wall muscle cell with six muscle arms (marked with asterisks) extending to the ventral nerve cord (lateral view). The micrograph shows fluorescence from both muscle and neuronal GFP reporters [*him-4p::MB::YFP* (muscle), *hmr-1b::DsRed2* (neuron), and *unc-129nsp::DsRed2* (neuron)]. All images are modified from WormAtlas (www.wormatlas.org). Photo credits: (A) D. Hall, (B) J. Maciejowski and E. J. Hubbard, and (C and D) WormAtlas.

The outer epithelial layer, the epidermis, of the embryo undergoes a series of cell fusions to make large multinucleate, or syncytial, epidermal cells. These cells secrete the cuticle, a protective layer of specialized extracellular matrix (ECM) consisting primarily of collagen, lipids, and glycoproteins (Chisholm and Hardin 2005; Page and Johnstone 2007). The cuticle determines the shape of the body and, through connection from the epidermis to muscle, provides anchoring points for muscle contraction (Figure 5A). The cuticle also serves as a model for ECM formation and function with molecules and pathways involved in cuticle biogenesis conserved in vertebrates (Page and Johnstone 2007).

**Figure 6:**
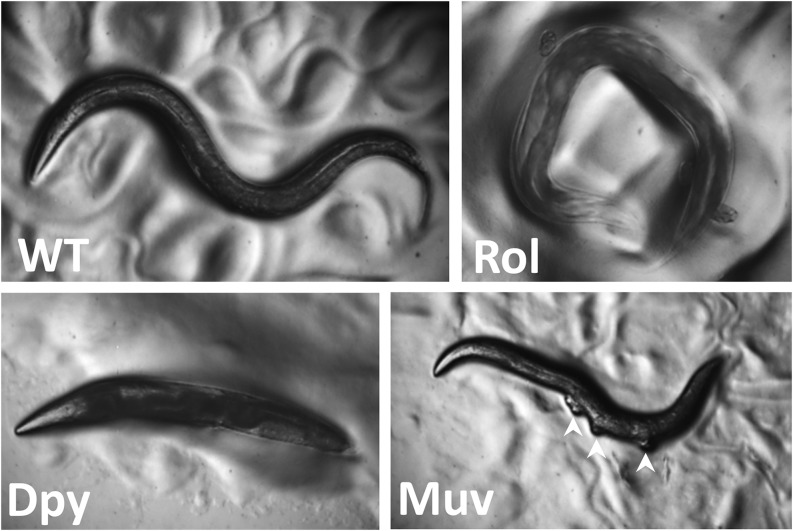
*C. elegans* mutant phenotypes. Wild-type animals (WT) are approximately 1 mm long with a smooth exterior, and they move in a sinusoidal pattern. Rolling (Rol) animals twist their body like a corkscrew and as a result often remain in the same region moving in a circular pattern. Dumpy (Dpy) animals are shorter than wild type. Multivulvae (Muv) hermaphrodites have protrusions along the ventral side (white arrowheads) where vulvae form but are not able to attach to the uterus. Strain sources: D. Eisenmann and A. Golden.

Mutations in several genes needed for cuticle formation produce visible phenotypes (Figure 6). Mutations in collagen genes can result in animals that move in a corkscrew fashion [the Roller (Rol) phenotype] or that have normal width but reduced length [the Dumpy (Dpy) phenotype]. Other mutations affect the struts formed between layers of the adult cuticle, resulting in fluid-filled blisters [the Blister (Bli) phenotype]. Still other mutations make the animals longer than normal [the Long (Lon) phenotype]. At the end of each larval stage (Figure 2), *C. elegans* sheds its cuticle and secretes a new one to accommodate the growing organism. Genes involved in cuticle formation are regulated so that the cuticle is reestablished after each molt.

Studying the epidermis has led to insights in early cell movements, wound healing, cell–cell fusions, and the establishment of epithelial layers in developing embryos (Chisholm and Hardin 2005; Podbilewicz 2006). As the “skin” of *C. elegans*, the epidermis is a model for the innate immune response to pathogens and for repair after a physical wound such as a needle puncture. The wounded epidermis upregulates both Ca^++^ signaling to direct actin polymerization for repair and innate immune signaling pathways to help promote survival after injury (Xu *et al.* 2012). The cell–cell fusion events leading to the multinucleate epidermis and genes important for this process have been studied (Podbilewicz 2006). This work has supported the idea that repression of fusion in some cells may be just as important for proper development as activation of cell fusion in other cells (Podbilewicz 2006).

### Muscles—controlling animal movement

Just interior and connected to the epidermis are four quadrants (95 cells) of body-wall muscles that run along the length of the body (Figure 3). The regular contraction and relaxation of the muscle cells leads to the “elegant” sinusoidal movement of the animal. These somatic muscles are striated (although, unusually, they are obliquely striated) and mononucleate (muscle cells do not fuse as they do in vertebrates) with multiple sarcomeres per cell (Moerman and Fire 1997). The innervation of these muscles is also unusual in that the muscle cells send extensions (“muscle arms”) to the ventral and dorsal cord to receive *en passant* synapses from the motor neurons instead of the more usual case of receiving axonal projections from motor neurons (White *et al.* 1986; Figure 5D).

Genetic studies of muscle led to the first cloning and sequencing of a myosin gene (*unc-54*; MacLeod *et al.* 1981), and this finding provided major insights into the structure of all myosins. *unc-54* and many other *unc* (uncoordinated) genes encoding proteins needed for muscle activity produce a “floppy paralytic” phenotype (Hodgkin 1983). The study of the assembly of sarcomeres into functional muscles and, in particular, the proteins mediating attachment to the plasma membrane has revealed many molecules in common with vertebrate focal adhesion complexes (Moerman and Williams 2006). Genetic screens designed to understand molecules involved in muscle contraction have also led to insights regarding muscle-wasting diseases such as Duchenne’s muscular dystrophy (Chamberlain and Benian 2000) and cardiomyopathies (Benian and Epstein 2011). In addition to the body-wall muscles, *C. elegans* has muscles that control eating (pharyngeal muscles), egg laying (vulval and uterine muscles and the contractile gonad sheath), mating (male-specific tail muscles), and defecation (enteric muscles).

### The digestive system—a model for organogenesis and pathogenesis

Food (bacteria) enters the anterior of the animals and passes through the pharynx, a two-lobed neuromuscular pump that grinds the food before it is passed on to the intestine for digestion (Figure 5C; Avery and You 2012). The pumping behavior of the animals depends on the availability and the quality of the food; for example, animals pump more when hungry and less when full (Avery and Shtonda 2003). Studying pharyngeal development has been a model for organogenesis, including how epithelial morphogenesis and cell-fate specification occur during development (Mango 2007). For example, the transcription factor PHA-4 plays a major regulatory role in the organ identity of the pharynx (Mango *et al.* 1994). Animals defective in PHA-4 function do not contain a pharynx and embryos that overexpress PHA-4 have more pharyngeal cells (Mango 2007). The vertebrate FoxA transcription factors are homologous to PHA-4 and are involved in gut development in many species (Carlsson and Mahlapuu 2002).

The *C. elegans* intestine is attached to the posterior pharynx and consists of 20 large, polyploid epithelial cells arranged in pairs that form a tube running the length of the animal. Intestinal development has been studied in detail (McGhee 2007). Presumably to handle the increasing demands of the growing animal, the intestinal cells undergo one round of nuclear division during the first larval stage and subsequent rounds of DNA replication, but not nuclear division, in the later larval stages (Hedgecock and White 1985). *C. elegans* has served as a model to study infection and response to infection by several different bacterial pathogens, microsporidia, and viruses that colonize the digestive system (Darby 2005; Balla and Troemel 2013; Diogo and Bratanich 2014).

### The nervous system—small yet complex

*C. elegans* has become an important model for the study of neurobiological questions. Researchers have identified genes and mechanisms needed for neuronal generation and specification, cell death, precursor migration, synapse formation, chemosensory and mechanosensory transduction, neuronal degeneration, neurite regeneration, and glial function (Driscoll and Chalfie 1992; Silhankova and Korswagen 2007; Hammarlund and Jin 2014; Shaham 2015). Researchers also have investigated a variety of behaviors, both simple and complex, including chemotaxis, thermotaxis, several responses to touch, male-specific mating rituals, social feeding, and both associative and nonassociative learning (Bargmann 2006; Hart 2006; Barr and Garcia 2006; Ardiel and Rankin 2010; Figure 4E). *C. elegans* also experiences periods of restful inactivity that are similar to aspects of mammalian sleep (Raizen *et al.* 2008).

The nervous system of the adult hermaphrodite has 302 neurons (Sulston and Horvitz 1977; White *et al.* 1986); that of the adult male has 383 neurons (Figure 4; WormAtlas.org). The majority of neuronal cell bodies are arranged in a few ganglia in the head, in the ventral cord, and in the tail (the specialized male tail has the majority of the extra neurons). Most of the neurons have a simple structure with one or two neurites (or processes) exiting from the cell body, but a few cells, such as the FLP and PVD mechanosensory neurons, have elaborately branched neurites (Dong *et al.* 2013). Except for sensory dendrites, which are often easy to identify, most neurites cannot be distinguished as axons or dendrites because they both give and receive synapses (although they are often referred to as “axons”). The neurites form synapses to each other in four major areas: the nerve ring (which encircles the pharynx), the ventral nerve cord, the dorsal nerve cord, and the neuropil of the tail. In addition to neurons, *C. elegans* has several glia-like support cells, which are primarily associated with sensory neurons, but are not as numerous as in vertebrates (Oikonomou and Shaham 2011).

Nerve conduction in *C. elegans* appears to be primarily passive. No sodium-dependent action potentials have been detected in neurons (Goodman *et al.* 1998) and the genome has no genes for voltage-gated sodium channels (Bargmann 1998). The absence of action potentials may be due to the very high membrane resistance. Indeed, many neurons are essentially isopotential; changes in voltage are experienced virtually instantaneously by the entire cell (Goodman *et al.* 1998), so action potentials may not be necessary (Lockery and Goodman 2009). Neurons express a wide variety of ion channels (Hobert 2013), including an unexpectedly large number of genes encoding potassium channels (Salkoff *et al.* 2005).

*C. elegans* neurons make more than 7000 chemical synapses and gap junction connections (White *et al.* 1986). Unlike in vertebrate nervous systems, *C. elegans* neurons do not send terminal branches with boutons to make synapses. Most of the connections are made *en passant* (side by side as neurites pass each other), although many bilaterally symmetrical neurons join their tips together with gap junctions at the midline and some motor neurons similarly join with homologs end to end. Nematodes are unusual in that motor neurons do not send processes that synapse onto muscle; instead muscles send cellular projections to motor neurons to receive synapses. Chemical synapses are identified in electron micrographs by presynaptic darkening and synaptic vesicles; postsynaptic specializations are not obvious (Figure 4D). *C. elegans* uses many of the most common neurotransmitters, including acetylcholine, glutamate, γ-amino butyric acid (GABA), dopamine, and serotonin and has several receptors for their detection (Hobert 2013). Gap junctions are detected in electron micrographs as parallel membranes in the closely apposed neurons. As with other invertebrates, gap junctions are formed from innexins (Starich *et al.* 2001). In addition to the chemical synaptic and gap junction connections, *C. elegans* neurons are modulated by numerous neuroendocrine signals (Li and Kim 2008). From the perspective of cellular and molecular detail, most of the problems of neurobiology can be studied in the worm.

In *C. elegans*, some individual neurons perform functions that would be performed by multiple neurons in vertebrates. For example, individual olfactory neurons express multiple G protein-coupled odorant receptors (Troemel *et al.* 1995; Wes and Bargmann 2001), rather than a single receptor as in vertebrates; each of the two mirror image bilateral ASE chemosensory cells respond to several different ions (Hobert 2010); and the connectivity of the touch receptor neurons suggests that their stimulation initiates several different activities (Chalfie *et al.* 1985). Thus, functions of multiple vertebrate sensory neurons are compressed into a single neuron in *C. elegans*. This multifunctionality (or “polymodality”) may be an evolutionary consequence of the small number of neurons in the *C. elegans* nervous system. Alternatively, multifunctionality may reflect a general feature of nervous systems that was revealed by the ability to do detailed single-cell analyses with this animal.

### Reproductive tissue—sex-specific anatomy

The *C. elegans* sexes display several obvious anatomical differences in the somatic gonad, secondary sexual structures, and body size (Figure 2, Figure 3, A and B). The somatic gonad is located in the center of the body alongside the intestine. In hermaphrodites the gonad consists of two mirror-image U-shaped tubes; in males the gonad consists of a single U-shaped lobe (Figure 3). Both gonads house the germline where the oocytes and sperm develop (Hubbard and Greenstein 2005). The somatic gonad and the germline develop together during larval stages until animals reach maturity at the young adult stage. A powerful advantage for studies of the *C. elegans* germline is that one can observe all stages of meiosis at once as the germline is a visible gradient of development (Hubbard and Greenstein 2005; Kimble and Crittenden 2005; Figure 5B). Studying the germline has been a model for meiosis, gamete development, fertilization, stem cell biology, and even tumor formation (Kimble and Crittenden 2005).

Secondary sexual mating structures are the vulva in hermaphrodites and the fan-shaped tail in males (Emmons 2005; Herman 2006). The vulva develops in the center of the epidermis on the ventral side of the hermaphrodite and is the conduit for sperm entry from the male and egg laying from the uterus (Sternberg 2005). Studying vulval morphogenesis has provided insights into signaling by the Notch, EGF, and Wnt pathways that coordinate the spatiotemporal development of the organ. Defects in these pathways can lead to animals with no vulva [the egg-laying defective (Egl) phenotype] or with many vulval-like protrusions [the multivulva (Muv) phenotype; Figure 6]. In many cases these animals cannot lay eggs (or mate), and their progeny develop internally, hatch within the hermaphrodite, and create the “bag-of-worms” phenotype whereby the larvae consume the mother (a process called endotokia matricidia). This phenotype, which also occurs when wild-type adult hermaphrodites are starved, has been used to identify numerous egg-laying defective mutants. For example, details of Ras GTPase/MAP Kinase signaling have been elucidated through the identification of mutants in enhancer/suppressor screens of vulval phenotypes (Sundaram 2013).

Adult males are thinner than hermaphrodites (due to their smaller gonad and the absence of developing embryos), and their tails are flattened into a fan of cuticular material with 18 projections of neurons and associated support cells called rays (Figure 3B). As with vulval development, the development of male tail structures and their associated muscles involves the coordinated action of multiple signaling pathways (Emmons 2005). Interestingly, the same signaling pathways are used in both males and hermaphrodites in the development of reproductive tissues (*e.g.*, Wnt signaling), yet these pathways produce very different sex-specific organs and structures (Emmons 2005).

The outward morphology of males and hermaphrodites is determined by a regulatory cascade that controls the transcription factor TRA-1 (Zarkower 2006). The activity of TRA-1 depends on the X-to-autosome (or X:A) ratio. In males, TRA-1 is inactive and leads to male fate and the production of sperm. In hermaphrodites, TRA-1 is active and leads to a female somatic fate and the formation of female gametes. *C. elegans* also has a dosage compensation system that downregulates expression of genes on the X-chromosome in hermaphrodites to equalize X-linked expression between the sexes. A sex-specific dosage compensation complex, analogous to the chromatin condensin complex used in mitosis and meiosis, decorates the X chromosomes in hermaphrodites and downregulates X-linked genes by 50% to equal the gene expression from the single X chromosome in males (Meyer 2005).

## The *C. elegans* Genome

*C. elegans* was the first multicellular eukaryotic organism to have its genome sequenced (*C. elegans* Sequencing Consortium 1998). As sequence information from additional *Caenorhabditis* species as well as more distantly-related nematodes has become available in the past decade, the information from *C. elegans* has provided the basis for rich comparative genomics studies (Coghlan 2005). The entire *C. elegans* genome is 100 Mb (*C. elegans* Sequencing Consortium 1998) and has 20,444 protein-coding genes (WormBase release WS245, October 2014). Both *C. elegans* sexes contain five autosomal chromosomes named linkage group (LG) I, II, III, IV, and V and the X chromosome. Individual genes of *C. elegans* are arranged in conventional eukaryotic fashion with 5′ untranslated regions, open reading frames (ORFs) containing exons and introns, and 3′ untranslated regions. Compared to vertebrate genes, *C. elegans* genes are relatively small with the average gene size of 3 kb due primarily to the presence of very small introns (Spieth and Lawson 2006; *C. elegans* genes also have many normal-sized introns). The chromosomes do not contain traditional centromeres; during mitosis the microtubule spindle attaches to more than one position along the chromosome (these attachments are said to be holocentric or polycentric). In fact, a specific sequence does not seem to be required for attachment since extrachromosomal DNA-containing transgenes can be inherited throughout many cell divisions.

The *C. elegans* genome has two unusual aspects: most protein-coding mRNAs are *trans*-spliced and some genes are organized in operons (Blumenthal 2005). *Trans*-splicing is the addition of one of two 22-nucleotide leader sequences (SL1 and SL2) at the 5′ end of mRNA. The leader sequence is believed to aid in translational initiation and, because SL1/2 sequences are known, can be used experimentally to identify the sequence at the 5′ end of mRNAs. Some *C. elegans* mRNAs are formed from multigenic transcripts with the first mRNA spliced to SL1 and subsequent mRNAs to SL2. The genes that code for these transcripts are closely spaced together in tandem and are transcribed under the control of a single promoter. These transcripts are similar to those produced by bacterial operons and code for gene products that are coexpressed (Blumenthal 2005). They differ, however, in that the processed transcripts in *C. elegans* generate multiple mRNAs. Past experiments have indicated that DNA is not methylated in *C. elegans*, but recent, higher resolution studies have suggested that some methylation does occur (Hu *et al.* 2015). Aspects of gene regulation such as transcription, translation, chromatin remodeling, and post-transcriptional modifications (ubiquitination, phosphorylation, histone methylation, and glycosylation) have all been studied using the genetic tools of *C. elegans*.

## *Caenorhabditis* Ecology and Evolution

In the wild, *C. elegans* is primarily restricted to temperate regions. In contrast, related species such as *C. briggsae* are considered “cosmopolitan” since they are found in many habitats (Kiontke and Sudhaus 2006). Although often mischaracterized as a soil nematode, larval and adult *C. elegans* have been routinely recovered from organic-rich garden soils, compost, and rotting fruit and plant stems, and only rarely from “wild” soil (Blaxter and Denver 2012; Félix and Duveau 2012). Compost-like locations are likely to be attractive to the animals because they are abundant in the bacterial food the animals need. Outside of rotting fruits and stems, *C. elegans* are usually found as dauer larvae, the dispersal form of the animal. Dauer larvae are unusual in that they engage in “nictating” behavior, wherein they stand on their tails and wave their heads in the air (Lee *et al.* 2011). This activity is thought to aid in attachment of the dauer larva to other invertebrate couriers, such as isopods, so the nematode can be dispersed from a depleted food source to a new food source (Croll and Matthews 1977). Recent work on nematode ecology has included studies on wild population structures and competition between *Caenorhabditis* species (Félix and Duveau 2012) and the effect of the richness of the bacterial biomass on nematode growth (Darby and Herman 2014). In addition, Rechavi *et al.* (2014) recently demonstrated that starvation in *C. elegans* leads to an upregulation of small RNAs that target nutrition genes and are inherited by multiple generations (“transgenerational inheritance”) providing a mechanism for memory of past environmental conditions.

**Figure 7:**
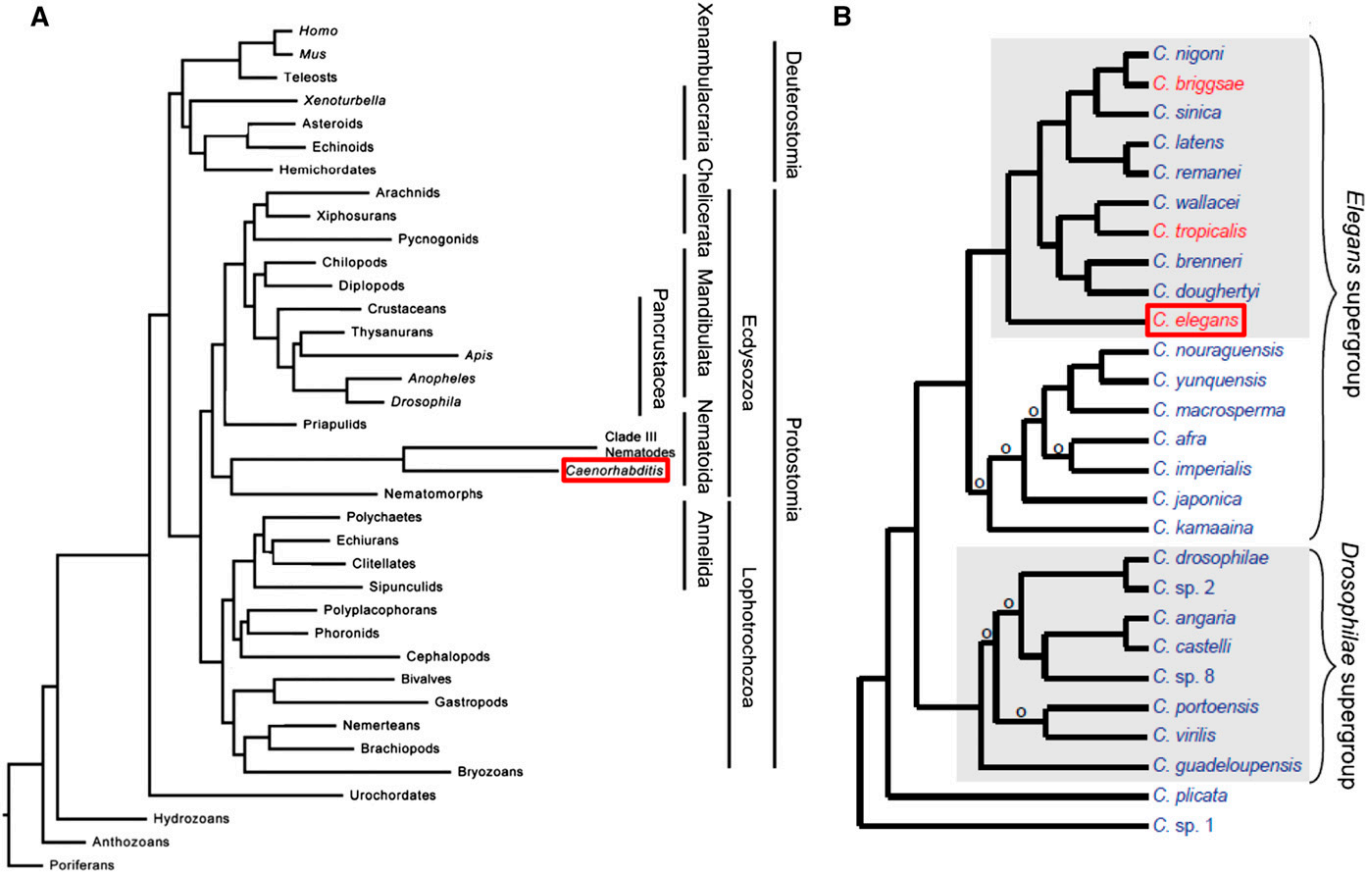
*Caenorhabditis* species in the animal kingdom. (A) Phylogenetic tree placing *Caenorhabditis* species (boxed in red) among metazoans based on sequence data from two ribosomal subunits, eight protein coding genes, and mitochondrial genomes. Image was modified from Bourlat *et al.* (2008). (B) Phylogenetic tree placing *C. elegans* (boxed in red) among named *Caenorhabditis* species grown in the laboratory. Species in red have hermaphrodites and males; species in blue have females and males. An ‘o’ denotes branches with low support. Image modified from Félix *et al.* (2014).

*Caenorhabditis* and other nematodes belong to the phylum Nematoda, which is part of a larger group of the clade Ecdysozoa (Figure 7A; Bourlat *et al.* 2008). This clade contains organisms that shed a cuticle by molting (or ecdysis) (Bourlat *et al.* 2008). Therefore, *C. elegans* are more related to *Drosophila* and other insects than to mollusks, earthworms, or humans. The *Caenorhabditis* genus is included in the order Rhabditida, itself part of the larger subclass Chromadoria (De Ley 2006). While all known *Caenorhabditis* species are free living, the Rhabditidae include animal and plant parasites, as well as free-living species that exist in a variety of terrestrial and aquatic ecosystems (Kiontke and Fitch 2005). As the ecology of *Caenorhabditis* is increasingly better understood, more species have been identified (Figure 7B; Félix *et al.* 2014). *C. elegans* has served as a rich starting place for comparative evolutionary studies among species. For example, developmental biologists use related nematode species to explore the changes in regulatory pathways and organogenesis that occur during evolution (Sommer 2005) and the frequent independent evolution of hermaphroditism (Figure 7B; Baldi *et al.* 2009).

The evolutionary relationship between *C. elegans* and parasitic nematode species has prompted research questions with direct human health relevance. Nematode parasites of humans cause major health problems, especially in less-developed nations. These parasites include hookworm and other soil-transmitted helminths that cause malnutrition and obstructive bowel disease, filarial nematodes such as *Onchocerca volvulus*, which causes river blindness, and *Brugia malayi*, which causes lymphatic filariasis (elephantiasis; Blaxter 1998). Plant parasitic nematodes cause significant crop damage (billions of dollars each year; Kandoth and Mitchum 2013) and animal parasites devastate domesticated animals (including heartworm in dogs and cats; McCall *et al.* 2008). *C. elegans* has played a critical role in elucidating the mode of action of anthelmintic drugs (Holden-Dye and Walker 2014). *C. elegans* may also prove useful in identifying new strategies to reduce or alleviate the action of parasitic nematodes, particularly since the emergence of resistance to current drugs (Holden-Dye and Walker 2014).

## Brief History of *C. elegans* Research and Key Discoveries

In Brenner’s original vision, detailed elucidation of the development and anatomy of *C. elegans* would serve as the foundation for the subsequent analysis of mutants. Both of these efforts were completed primarily at the LMB. The transparency of the animal allowed John Sulston, Robert Horvitz, Judith Kimble, David Hirsh, and Einhard Schierenberg to describe every cell division starting with the single-celled zygote and ending with the adult male and hermaphrodite (Sulston and Horvitz 1977; Kimble and Hirsh 1979; Sulston *et al.* 1980, 1983). These efforts produced the first and only entire cell lineage of any multicellular organism. During this same time, John White, Sydney Brenner, Donna Albertson, Eileen Southgate, Sam Ward, and Nichol Thomson described the anatomy and connectivity of all 302 neurons of the adult hermaphrodite (Ward *et al.* 1975; Albertson and Thomson 1976; White *et al.* 1976, 1986). These projects set a standard for completeness in the understanding of the animal that has been a hallmark of *C. elegans* research. Such completeness was also seen in the sequencing of the *C. elegans* genome (*C. elegans* Genome Consortium 1998), the description of the wiring diagram of the adult male (Jarrell *et al.* 2012), and development of genome-wide feeding RNAi experiments (Fraser *et al.* 2000; Kamath *et al.* 2001). Sydney Brenner, Robert Horvitz, and John Sulston were awarded the 2002 Nobel Prize in Physiology or Medicine in part for the significance of the lineage project as a platform for discovery of genes that orchestrated developmental decisions.

Since work on *C. elegans* genetics began in earnest during the 1970s, this animal has proven fruitful for making general discoveries about cell and developmental biology (Table 2). These findings have helped us understand molecular genetic mechanisms in all animals. Evolution has maintained thousands of conserved genes that play similar, or in some cases nearly identical, functions in nematodes and other animals including humans (Carroll *et al.* 2004). For example, the vertebrate apoptosis regulator Bcl-2 can functionally substitute for its *C. elegans* ortholog *ced-9* (Hengartner and Horvitz 1994). Thus, discoveries with direct relevance to understanding all animals were made possible by what initially appeared to be an ambitious and esoteric undertaking to define in detail the structure and genetics of an apparently simple animal.

**Table 2:** Selected discoveries in *C. elegans* research

Year	Discovery	References
1974	Identification of mutations that affect animal behavior	Brenner 1974 PMID: 4366476; Dusenberry *et al.* 1975 PMID: 1132687; Hart 2006
1975	First description of mutations that affect thermotaxis and mechanotransduction	Hedgecock and Russell 1975 PMID: 1060088;
		Sulston *et al.* 1975 PMID: 240872; Chalfie and Sulston 1981 PMID: 7227647; Mori and Ohshima 1995, PMID: 7630402
1977	First cloning and sequencing of a myosin gene	Macleod *et al.* 1977 PMID: 909083
1977	Genetic pathways for sex determination and dosage compensation described	Hodgkin and Brenner 1977 PMID: 560330; Meyer 2005 PMID: 18050416; Zarkower 2006 PMID: 18050479
1981	Identification of mutations affecting touch sensitivity	Sulston *et al.* 1975 PMID: 240872; Chalfie and Sulston 1981 PMID: 7227647
1981	First germline stem cell niche identified	Kimble and White 1981 PMID: 7202837; Kimble and Crittenden 2005 PMID: 18050413
1983	Notch signaling, presenilins, ternary complex, and lateral inhibition roles in development described	Greenwald *et al.* 1983 PMID: 6616618; Levitan and Greenwald 1995 PMID: 7566091; Petcherski and Kimble 2000 PMID: 10830967; Greenwald and Kovall 2012 PMID: 23355521
1983	First complete metazoan cell lineage	Sulston and Horvitz 1977 PMID: 838129; Kimble and
		Hirsh 1979 PMID: 478167; Sulston *et al.* 1983 PMID: 6684600
1983	Discovery of apoptosis (cell death) genes	Hedgecock *et al.* 1983 PMID: 6857247; Ellis and Horvitz 1986 PMID: 3955651; Yuan and Horvitz 1992 PMID: 1286611; Yuan *et al.* 1993 PMID: 8242740; Conradt and Xue 2005 PMID: 18061982
1984	Identification of heterochronic genes	Ambros and Horvitz 1984 PMID: 6494891; Slack and Ruvkun 1997 PMID: 9442909
1986	First complete wiring diagram of a nervous system	White *et al.* 1986 PMID: 22462104; Jarrell *et al.* 2012 PMID: 22837521; White 2013 PMID: 23801597
1987	Discovery of the first axon guidance genes	Hedgecock *et al.* 1987, 1990 PMID: 3308403 PMID: 2310575; Culotti 1994 PMID: 7950328
1987	Identification of role of Notch signaling in embryonic blastomeres	Priess *et al.* 1987 PMID: 3677169; Priess 2005 PMID: 18050407
1988	Discovery of *par* genes, whose products affect the asymmetric distribution of cellular components in embryos	Kemphues *et al.* 1988 PMID: 3345562; Gönczy and Rose 2005 PMID: 18050411
1988	Identification of the first LIM and POU homeodomain transcription factors	Way and Chalfie 1988 PMID: 2898300; Finney *et al.* 1988 PMID: 2903797; Hobert 2013 PMID: 24081909
1990	First description of a role for RAS signaling function in metazoan development	Beitel *et al.* 1990 PMID: 2123303; Han and Sternberg 1990 2257629; Sternberg 2005 PMID: 18050418; Sundaram 2013 PMID: 23908058
1993	Demonstration of a role for insulin pathway genes in regulating lifespan	Friedman and Johnson 1988 PMID 8608934; Kenyon *et al.* 1993 PMID: 8247153; Kimura *et al.* 1997 PMID: 9252323; Collins *et al.* 2007 PMID: 18381800
1993	Identification of genes for conserved synaptic functions	Gengyo-Ando *et al.* 1993 PMID: 8398155; Richmond *et al.* 1999 PMID: 10526333; Richmond 2007 PMID: 18050398
1993	First microRNA (*lin-4*) and its mRNA target (*lin-14*) described	Lee *et al.* 1993 PMID: 8252621; Wightman *et al.* 1993 PMID: 8252622; Vella and Slack 2005 PMID: 18050425
1993	Identification of nonsense-mediated decay genes	Pulak and Anderson 1993 PMID: 8104846; Hodgkin 2005 PMID: 18023120
1994	Introduction of GFP as a biological marker	Chalfie *et al.* 1994 PMID: 8303295; Boulin *et al.* 2006 PMID: 18050449
1994	First demonstration of specific olfactory receptor/ligand pair	Sengupta *et al.* 1994 PMID: 8001144; Bargmann 2006 PMID: 18050433
1998	First metazoan genome sequenced	*C. elegans* Sequencing Consortium 1998 PMID: 9851916; Schwarz 2005 PMID: 18023117
1998	Discovery of RNA interference (RNAi)	Fire *et al.* 1998 PMID: 9486653
2000	Conservation and ubiquity of miRNAs	Pasquinelli *et al.* 2000 PMID: 11081512
2000	Development of genome-wide RNAi screening/first full genome-wide profiling of gene function	Fraser *et al.* 2000 PMID: 11099033; Kamath *et al.* 2001 PMID: 11178279
2000	Transgenerational inheritance and its mediation by piRNA	Grishok *et al.* 2000 PMID: 10741970; Ashe *et al.* 2012 PMID: 22738725
2002	First cytoplasmic polyA polymerase (*gld-2*) discovered	Wang *et al.* 2002 PMID: 12239571; Kimble and Crittenden 2005 PMID: 18050413
2005	First full-genome RNAi profiling of early embryogenesis	Sönnichsen *et al.* 2005 PMID: 15791247
2005	First use of channelrhodopsin optogenetics in an intact animal	Nagel *et al.* 2005 PMID: 16360690
2011	Discovery of first nematode viruses	Félix *et al.* 2011 PMID: 21283608

Genetic screens in *C. elegans* have yielded a number of first discoveries of genes and pathways that play important roles in all animals. These discoveries include key genes regulating apoptosis (programmed cell death) (Hedgecock *et al.* 1983; Ellis and Horvitz 1986), the Ras and Notch signaling pathways (Priess 2005; Greenwald and Kovall 2013; Sundaram 2013), synaptic function (Gengyo-Ando *et al.* 1993; Richmond *et al.* 1999), axon pathfinding (Hedgecock *et al.* 1987, 1990), longevity (Kenyon *et al.* 1993; Kimura *et al.* 1997), and developmental timing (the heterochronic genes) (Ambros and Horvitz 1984). The study of the heterochronic genes yielded the first small regulatory RNA, the microRNA (miRNA) product of the *lin-4* gene (Lee *et al.* 1993). Robert Horvitz’s 2002 Nobel Prize was awarded in part to recognize the broad significance of the genetic mechanisms of apoptosis, and Gary Ruvkun and Victor Ambros shared the 2008 Albert Lasker Award for Basic Medical Research in recognition of the general importance of miRNA.

*C. elegans* researchers also made important technical discoveries that were subsequently adapted and applied to other biological systems. For example, early gene-cloning efforts helped advance techniques for defining and assembling overlapping (contiguous) clones from animal genomes, which led to *C. elegans* being the first metazoan to have its entire genome sequenced in 1998 (*C. elegans* Genome Consortium 1998). Some of these strategies were also employed in early human genome sequencing efforts. Another discovery that led to a novel technique with broad biological impact was gene silencing by RNA interference (RNAi) (Fire *et al.* 1998). This powerful technique allows researchers working with many organisms to silence the expression of any gene and earned Andrew Fire and Craig Mello the 2006 Nobel Prize in Physiology or Medicine (Fire 2007; Mello 2007). Finally, the development of green fluorescent protein (GFP) as a biological marker (Chalfie *et al.* 1994) for which Marty Chalfie shared the 2008 Nobel Prize in Chemistry grew directly from his interest in characterizing gene expression in live (and transparent) *C. elegans*. Now GFP and other fluorescent proteins are widely-used biological tools.

## The *C. elegans* Community

Having mentioned all the biological reasons that *C. elegans* provides outstanding opportunities to study biological questions and all the achievements that past studies have accomplished, we would be remiss if we did not mention one other reason why the field has flourished: the community of *C. elegans* researchers. Our field has had a long tradition of openness and sharing of reagents and ideas. This openness was first encouraged by the publication of the *C. elegans* newsletter, *The Worm Breeder’s Gazette*. The *Gazette* was started by Bob Edgar at the University of California, Santa Cruz so *C. elegans* researchers could tell each other about their research. Often work was described in the *Gazette* many months before it was officially published. For example, the first description of GFP as a biological marker (in the October 1993 issue of the *Gazette*) preceded the “official” publication by 5 months (Chalfie *et al.* 1994). The *Gazette*, now entirely online, is published as part of WormBook and is an excellent resource for new methods and helpful hints to aid *C. elegans* research.

The second person to greatly support and promote a community spirit among *C. elegans* researchers was John Sulston. When John began determining the physical map of the *C. elegans* genome with Alan Coulson, one of his goals was to promote sharing. He did this by not having any independent research of his own (taking away even a hint that he might be a competitor) and by providing a service whereby any researcher could get large amounts of DNA on either side of a cloned fragment or in the region of a mapped gene. As a result, researchers did not hoard their DNA before publication and investigators benefited by having clones of entire genes. The mapping project was able to link the physical (DNA) and genetic (recombination) maps. Consequently, several collaborations were initiated because scientists shared their data before publication (*e.g.*, Savage *et al.* 1989; Miller *et al.* 1992). As the mapping project turned to the sequencing of the *C. elegans* genome, John made sure that the openness continued; results of the sequencing project were made available daily. This same approach was used when John and Alan along with Bob Waterston and many others turned to sequencing the human genome.

A field with many thousands of researchers cannot be as closely associated as in the early days when virtually all researchers knew one another because they were Sydney Brenner's F1 and F2 progeny. Nonetheless, the *C. elegans* field continues to share resources and information. The many free online resources (Table 1), the *Caenorhabditis* Genetics Center, and the many large, community projects (*e.g.*, ModEncode Project, National Bioresource Project for the Experimental Animal *C. elegans*, and *C. elegans* Gene Knockout Consortium) show that the sharing spirit continues. This spirit and the general enthusiasm of *C. elegans* researchers are also evident at the biennial *C. elegans* and the many alternate-year special topics and local meetings. *C. elegans* has also proven a useful system for undergraduate and even high school education. The field continues to grow and to share.

Looking to the future, *C. elegans* will continue to be an important source of scientific discoveries. Many of the reasons for past successes will aid future research, especially the ability to look at individual cells within the context of the entire animal. Soon genome editing should generate loss-of-function alleles and transcriptionally and translationally tagged reporters for every gene in the genome allowing structure/function studies of all the genes and their encoded products. In addition, analysis of the ever-increasing collection of regulatory RNAs certainly will add to our understanding of the development and adaptability of *C. elegans*. Continued use of optogenetics on larger and larger sets of neurons and the analysis of the functional expression of neurotransmitters, neuropeptides, and their receptors and of gap junction proteins is likely to produce the first integrated view of the working of a complete nervous system. Investigations using *C. elegans* promise to generate insights into new areas, such as host–pathogen interactions, synthetic biology, and ecology. Finally, we are confident that new and unexpected discoveries made in *C. elegans*, as they have done so often in the past, will change our views of how organisms develop, live, and age. We invite you to join us to be part of this exciting future.
